# Raman spectroscopy mapping of changes in the organization and relative quantities of cell wall polymers in bast fiber cell walls of flax plants exposed to gravitropic stress

**DOI:** 10.3389/fpls.2022.976351

**Published:** 2022-08-22

**Authors:** Anne-Sophie Blervacq, Myriam Moreau, Anne Duputié, Isabelle De Waele, Ludovic Duponchel, Simon Hawkins

**Affiliations:** ^1^Université de Lille, Sciences et Technologies, CNRS, UMR 8576 - UGSF - Unité de Glycobiologie Structurale et Fonctionnelle, Lille, France; ^2^Université de Lille, Sciences et Technologies, CNRS, UMR 8516 - LASIRE - Laboratoire de Spectroscopie pour les Interactions, la Réactivité et l’Environnement, Plateforme FT-Raman, Lille, France; ^3^Université de Lille, Sciences et Technologies, CNRS, UMR 8198 - EEP - Evo-Eco-Paléo, Lille, France; ^4^Université de Lille, Sciences et Technologies, CNRS, UMR 8516 - LASIRE – Laboratoire de Spectroscopie pour les Interactions, la Réactivité et l’Environnement, Lille, France

**Keywords:** bast fibers, cell wall, abiotic stress, Raman spectroscopy, chemometric, flax (*Linum usitatissimum* L.)

## Abstract

Flax is an important fiber crop that is subject to lodging. In order to gain more information about the potential role of the bast fiber cell wall in the return to the vertical position, 6-week-old flax plants were subjected to a long-term (6 week) gravitropic stress by stem tilting in an experimental set-up that excluded autotropism. Stress induced significant morphometric changes (lumen surface, lumen diameter, and cell wall thickness and lumen surface/total fiber surface ratio) in pulling- and opposite-side fibers compared to control fibers. Changes in the relative amounts and spatial distribution of cell wall polymers in flax bast fibers were determined by Raman vibrational spectroscopy. Following spectra acquisition, datasets (control, pulling- and opposite sides) were analyzed by principal component analysis, PC score imaging, and Raman chemical cartography of significant chemical bonds. Our results show that gravitropic stress induces discrete but significant changes in the composition and/or spatial organization of cellulose, hemicelluloses and lignin within the cell walls of both pulling side and opposite side fibers.

## Introduction

In order to optimize photosynthetic capacity, higher land plants show negative geotropism leading to the development of a stable architecture that enables them to grow vertically (Bastien et al., 2015; Moulia et al., 2019). During their life, plants are constantly exposed to a changing environment with variations in temperature, rainfall and wind that modify the vertical position of the stem. When climatic conditions (especially rain and wind) are sufficiently severe plants undergo the phenomenon known as lodging in which the stems are bent parallel to the ground surface. In cereal crops such as rice and wheat, lodging makes harvesting difficult and has led to the selection of dwarf varieties (Vera et al., 2012).

Flax (*Linum usitatissimum* L.) is an important natural fiber source of considerable economic importance (Gorshkova and Morvan, 2006) that has been used by humans since the Paleolithic (Kvavadze et al., 2009). This species is grown for both linen textile fibers (Day et al., 2005; Chen and Yu, 2011) and for seeds (Singh et al., 2011). Nowadays, flax is also included in bio-composite raws, thermal insulation of buildings and medical applications (Bagheri et al., 2015). Like many other annual crops, flax plants are also subjected to lodging which negatively affects plant harvesting and fiber yields (Vera et al., 2012; Goudenhooft et al., 2019). Given its economic importance, information about how this species reacts to lodging is therefore of both practical and fundamental interest.

**FIGURE 1:**
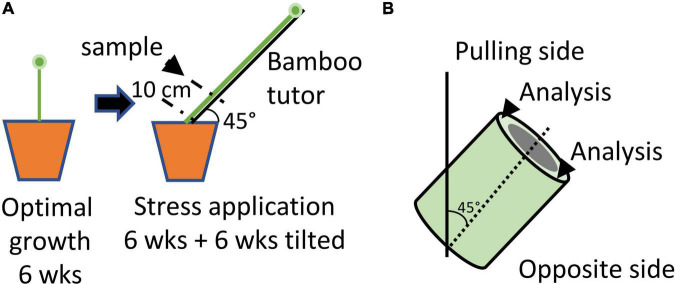
Schematic diagram illustrating the growing conditions used to induce gravitropic stress in flax stems. **(A)** Protocol. Plants were grown under optimal conditions for 6 weeks before being attached to bamboo tutors inclined at an angle of 45° for a further 6 weeks. Attached plants were unable to recover to the vertical position. Samples (stem section) were collected at 10 cm above the cotyledon scars. **(B)** The upper side of the stem sample was considered as the “pulling side” and the lower side as the “opposite side”.

Recent studies (Ibragimova et al., 2017; Goudenhooft et al., 2019; Beaugrand et al., 2022) have shown that when pot-grown plants are placed horizontally, they are able to return to the vertical position after 4 days. Both histochemistry and FTIR analysis of these plants suggested changes in the structure of bast fibers from tilted plants compared to those from non-tilted plants. In the model system developed by Ibragimova and colleagues (Ibragimova et al., 2017) young flax plants are exposed to a short gravitational stress over a 4-day period by inclining pots at 90°. Since the return to the vertical position in plants normally involves both gravitropism and autotropism (Moulia et al., 2021) it is likely that this is also the case for the observed recovery of tilted flax plants. In order to separate these two factors, we developed a model system whereby 6-week-old plants were inclined at 45° for a period of 6 weeks (Figure 1) thereby eliminating, or greatly reducing the autotrophic effect. This system is also a more accurate representation for lodged flax plants in the field since, depending upon climatic conditions, stems can quickly become entwined and covered by mud thereby hindering a rapid return to the vertical position (Goudenhooft et al., 2019).

In order to obtain detailed information about potential changes to cell wall composition in flax fibers during gravitropism we decided to use an imaging approach based on Raman micro-spectroscopy. Vibrational spectroscopy techniques such as FT-IR and Raman have been widely used to characterize cell wall composition in plants. For example, FT-IR spectroscopy has been shown to be a powerful tool for the global investigation of cell wall polymer composition (Naumann and Polle, 2006; Cai et al., 2011; Matos et al., 2013) as well as for monitoring cell wall plasticity (Largo-Gosens et al., 2014) or distinguishing *Arabidopsis* genotypes and mutants (Mouille et al., 2003; da Costa et al., 2014). Similarly, Raman spectroscopy has often been used to characterize poplar wood in the context of renewable material studies using an interdisciplinary approach combining biology, chemistry and physics (Gierlinger et al., 2008, 2012, 2013; Gierlinger, 2014, 2018; Felhofer et al., 2020).

In this paper we report the use of a Raman vibrational spectroscopy approach combining statistical analysis (principal component analysis: PCA), principal component (PC) score imaging and chemical cartography of statistically significant bands to provide detailed spatial information at the micro-scale level of modifications in cell wall polymers of fibers from tilted vs. non-tilted plants. Our results provide novel information on the extent and complexity of cell wall modifications induced by gravitational stress in flax plants.

## Materials and methods

### Plant material and gravitropic stress induction

Seeds of fiber flax (*Linum usitatissimum* L., var. Diane) were germinated in an 11 x 11 cm pot and plants grown in a greenhouse under natural light conditions with daily watering. Plants exposed to gravitropic stress were grown for 6 weeks before being attached to a bamboo pole oriented at 45° to the vertical position (Figure 1). Plants were maintained attached to the pole for a further 6 weeks before harvest. Control plants were unattached and harvested at the same time as stressed plants (12 weeks after germination).

### Sample fixation and section preparation

Stem samples (1-cm-long) were collected from the middle region of the stem (10 cm above the cotyledon scar) from ten individual control and ten individual stressed plants. For morphometric analyses samples were fixed in EtOH (70% w/v) for a minimum of three days at 4°C before being dehydrated through a graded EtOH/Histoclear series and embedded in Paraplast Plus. Stem transversal sections (10 μm) were obtained with a Leica RM65 microtome equipped with an S65 metal blade. Sections were mounted on glass slides and stained with Toluidine Blue-O (TBO) prior to observation with an Olympus BH-2 light microscope. For Raman microspectroscopy, samples were embedded in PEG (polyethylene glycol) 1,500, as previously described (Gierlinger, 2014). Transversal sections (30 μm) were obtained with a Leica RM2065 microtome for Raman focusing. PEG was removed by multiple water baths and sections were air dried prior to mounting in bidistilled water with a coverslip sealed by nail polish to avoid desiccation. Standard glass slides (superfrost™) and glass coverslips were used.

### Morphometric data treatment

Measures were acquired with FIJI free software^1^. The criteria selected were: surface of the lumen excluding the cell wall, longest lumen diameter excluding the cell wall (as fibers are usually oval), cell wall thickness measured from the middle lamella to the lumen, and surface ratio [lumen surface/fiber total surface, in%]. The surface of the fibers was measured using FIJI tool positioned all around the fiber at the middle lamella location. Measurements were made on three categories of bast fibers: (i) bast fibers from control plant sections, (ii) bast fibers located in the “pulling side” (top side) of tilted plants and (iii) bast fibers from the “opposite side” (bottom side) of tilted plants. Stems were collected from 3 control to 3 tilted plants. A total of 50 to 150 measures were performed for each criterion and were acquired from 3–5 cross sections/stem sample.

Preliminary statistical analyses (Shapiro-Wilk test) showed that data sets were not normally distributed nor homoscedastic and the Mood’s test was therefore applied to compare the median of each criterion (Mood, 1954). *P*-values were adjusted by the FDR procedure (Benjamini and Hochberg, 1995) to account for multiple testing.

### Raman microspectroscopy

#### Spectral acquisition

**FIGURE 2:**
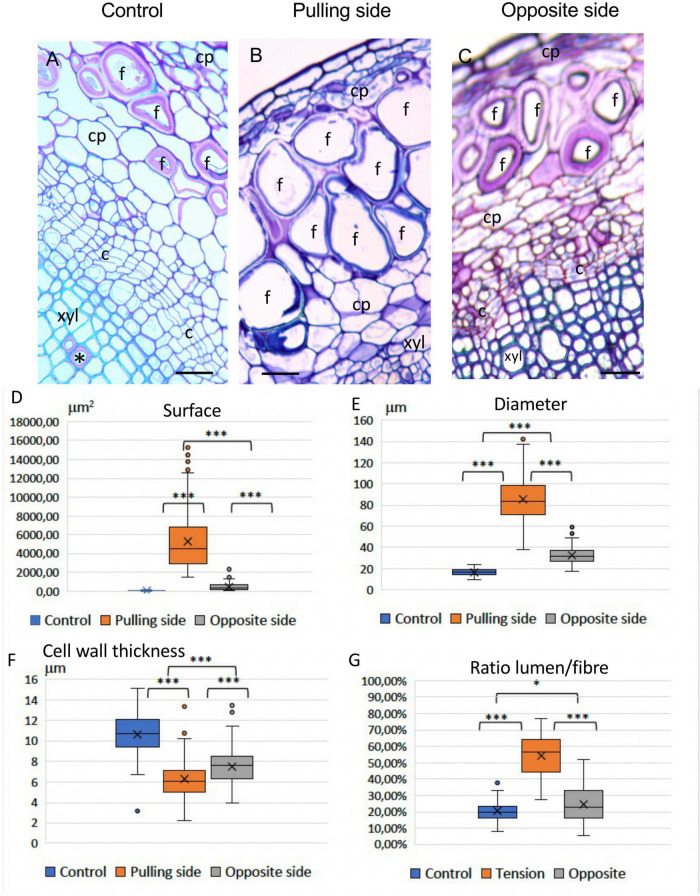
**(A)** Average spectra of bast fibers from control conditions (blue), and stressed conditions: pulling side (red) and opposite side (green). Acquisition was performed in the plant cell wall fingerprint range (300: 1,800 cm^–1^). Spectra were treated for the baseline, then aligned on the cellulose band (379 cm^–1^). Main chemical bonds were assigned to cell wall polymers (Black asterisk: cellulose, Red asterisk: lignin, Green asterisk: xylan and xyloglucan). **(B)** PC1 (92.31%) loading vector. Bands were assigned to cell wall polymers: Black asterisk: cellulose, Red asterisk: lignin, Green asterisk: xylan and xyloglucan.

Analyses were performed on a LabRam HR-Evolution (Horiba scientific). Average spectra were obtained for each bast fiber category (control, pulling side and opposite side) and normalized on the 379 cm^–1^ peak assigned to cellulose (Figure 2A). For excitation, 515 nm radiation from a Cobolt laser diode with a laser power of 10 mW incident on the sample was used. The spectrophotometer has an entrance slit of 100 μm, and is equipped with a 600 lines/mm^–1^ grating that permits to achieve a spectral resolution of 1.8 cm^–1^ per pixel. The Raman scattered light was detected with a CCD camera operating at 200K. An Olympus × 100/0.9 objective focused the laser light on the samples with a laser spot of 0.8 μm. For spatially resolved measurements, an x–y motorized stage (Merzhäuser) with a minimum possible step size of 0.1 μm was used. The maps were recorded with spatial resolution of 1 μm in both (x and y) directions. The z direction was fixed during the map recording. For wavenumber calibration, a silicium plate was used as a reference for subsequent data pre-processing. Spectra were acquired at 515 nm, with 5 min bleaching, 100 accumulation/sec, from 300 to 1,800 cm^–1^ corresponding to the plant cell wall fingerprint region. All statistical analyses were performed with MATLAB software (The MathWorks Inc., Natick, MA, United States).

#### Principal component analysis and imaging

Principal component analysis (PCA) was used to analyze high-dimensional spectral data sets and generate 2D images (PC score images) as described previously (Moncayo et al., 2018). Briefly, the Raman spectral data can be considered as a 3-D matrix (a hyperspectral cube) that contains both spatial (X and Y dimensions) and Raman spectral (Z dimension) data for each pixel. For PCA analysis this data set is “unfolded” to generate a 2-D matrix of dimension N (Number of pixels × Number of wavelengths). PCA analysis of the unfolded matrix is then performed to generate a selected number of PCs that explain the majority of the variability. PCA was applied to the first derivative spectral data according to the Savitzky-Golay algorithm (Savitzky and Golay, 1964; Spectra, 1985; Robert et al., 1996). Spectra were normalized with Surface-Area = 1 model. Each PC is associated with a scores matrix that can be used to plot a 2D image (a PC score image) and a loadings matrix that provides the loading vectors. In the PC image, pixels of similar values (scores) are similar in terms of the different components (here, Raman spectral peaks corresponding to different cell wall polymers) contributing to the loading vector of the PC considered. A Whittaker curve [log (eigenvalues) = principal component numbers] was calculated in order to determine the number of eigenvalues necessary to explain around 95% of the variance (Zhang et al., 2017; Vallejo-Pérez et al., 2021). In our case the curve indicated that the first six PCs should be imaged.

#### Raman chemical imaging

Raman chemical imaging was performed using major individual peak wavenumbers identified in the PC loading vectors. To enable comparison of different chemical images, a ratio was used based on the intensity of the reference cellulose peak at 379 cm^–1^.

## Results

### Long-term tilting induces significant changes in bast fiber morphology

**FIGURE 3:**
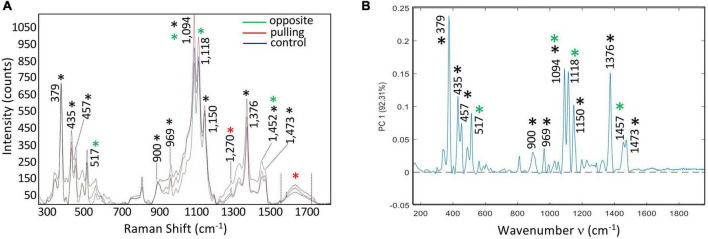
Effects of a gravitropic stress on bast fibers (BFs). **(A)** 12-week-old control stem showing appearance of BFs in stem sections stained with Toluidine Blue O (TBO). Rare G-layers (*) are observed in some vessels (wood). **(B)** Pulling side of stressed sample, showing greatly altered BFs. **(C)** Opposite side (of the same stem), shows BFs similar to the control **(A)** c: cambium; cp: cortical parenchyma; f: bast fibers; xyl: secondary xylem (wood). Bar = 50 μm. **(D–G)** Boxplots of morphometric data, Lumen surface **(D)**, Lumen Diameter **(E)**, Cell wall thickness **(F)**, and Ratio Lumen surface/Total surface of the fiber **(G)**. Mood’s test was applied and *P*-value was adjusted according to the FDR procedure. All pairwise *P*-values were < 0.001, ^***^ (see section “Materials and methods”).

Light microscopy of TBO-stained stem cross-sections from 12-week-old flax plants revealed that bast fibers from the pulling (upper) side of tilted plants showed a very different morphology compared to control bast fibers (Figure 3). Pulling side fibers show a morphologically altered shape (Figures 3B,D–G), have a larger lumen surface and a thinner deformed cell wall. The morphology of bast fibers from the opposite side seemed similar to the control according to morphology (size, lumen surface) and color (after TBO staining) (Figures 3C,D–G). Some gaps between different cell wall layers, or at the corner of cells could also be observed in altered fibers (Supplementary Figure 1).

Statistical analysis (Table 1) of morphometric criteria (Figure 3D: lumen surface, Figure 3E: lumen diameter, Figure 3F: cell wall thickness, Figure 3G: ratio [lumen surf/total fiber surf]) revealed significant differences between all three categories of bast fibers according to the Mood’s test. While differences were expected between pulling side and control fibers, the fact that opposite fiber morphology is also significantly altered is of interest given their visual similarity with control fibers. Taken together, our results showed that long-term bending induced modifications in bast fiber morphology. In addition to the observed morphological changes, pulling side fiber cell walls were colored dark blue/violet with TBO in contrast to the pink color of control and pulling side fibers suggesting changes in cell wall pH and/or composition (O’Brien et al., 1964).

**TABLE 1:** Morphometric parameters of control and gravitropic stressed fibers.

	Lumen surface (μm^2^)	Lumen diameter (μm)	Cell Wall Thickness (μm)	Ratio Lumen/Fiber %
**Control**	85.9 ± 31.48 ***	16.30 ± 3.59 ***	10.65 ± 2.02 ***	20.71 ± 7.06 *
**Pulling side**	5295.19 ± 3046,28 ***	85.50 ± 20.74 ***	6.31 ± 1.52 ***	54.24 ± 12.29 ***
**Opposite side**	487.19 ± 375.98 ***	32.55 ± 8.11 ***	7.50 ± 1.63 ***	24.70 ± 10.32 *

Pulling side and opposite side fibers were compared to control fibers using Mood’s test and P-value adjusted according to the FDR procedure, ***, * indicate P-values < 0.001, and 0.05, respectively.

### Raman principal component score imaging reveals statistically significant differences between the spatial distribution of cell wall polymers in control and tilted bast fibers

In order to obtain a more detailed understanding of the potential changes in cell wall structure associated with the altered morphology induced by bending we used Raman micro-spectroscopy. A comparison of the average spectra of the three samples (control, tilted-pulling side, tilted-opposite side; Figure 2A) showed that globally they were very similar. Although differences were observed in the relative intensities of the assigned peaks the similarity of the three spectra would suggest that the overall fiber cell wall chemical composition is only slightly modified by tilting despite the clear differences in morphology. To obtain further information, we used two complementary imaging approaches based on multivariate data analysis: (1) Principal Component score imaging and (2) Raman chemical mapping of significant loading vector bands.

When the PCA multivariate approach was used to analyze the combined hyperspectral data obtained from control, pulling-side and opposite-side bast fibers we observed that the first six PCs explained 95.01% of the total variance. The first principal component (PC1) explained 92.31% of the total variance of the three acquired hyperspectral data sets (Figure 2B). Further inspection of this first component shows that it is very similar, but not identical, to the average spectra of the three samples. All of the peaks assigned to cellulose and hemicellulose in the average spectra were also present in the PC1 loading vector, but at different relative intensities. For example, the 1,094 and 1,118 cm^–1^ bands, respectively assigned to cellulose/xyloglucan and xylan showed reduced peak height compared to the 379 cm^–1^ band assigned to cellulose. The 1,270 and 1,599 cm^–1^ peaks related to lignin were also absent in the PC1 loading vector. The high similarity between the average spectra and the PC1 loading vector, together with the fact that no negative contributions are observed in PC1 would suggest that this first main component corresponds mainly to an overall variation in the spectrum. The observed differences are not biologically related but rather result from physical variations such as laser intensity or small variations in the surface flatness of the sections. Such an interpretation is in agreement with the observed similarity of the normalized spectra from the three samples (Figure 2A).

**FIGURE 4:**
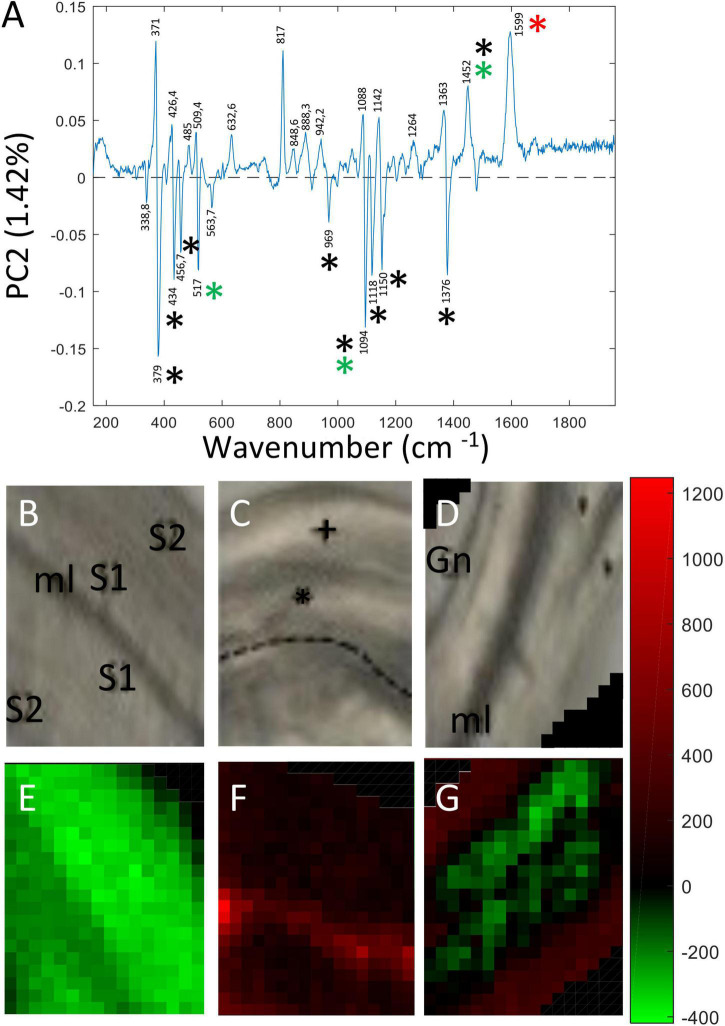
PC score imaging of PC2 (1.42%). **(A)** Loading vector of PC2. Peaks making positive contributions concerned chemical bonds related to lignin (1,270; 1,599 cm^–1^), cellulose/xyloglucan (1,452 cm^–1^). Peaks making negative contributions were mainly assigned to cellulose (379; 435; 457; 1,094; 1,150; 1,376 cm^–1^), xylan (1,118 cm^–1^) and xyloglucan (517 cm^–1^). Only major contributions (positive or negative) are considered. Black asterisk: cellulose, Red asterisk: lignin, Green asterisk: xylan and xyloglucan. Blue asterisk: pectin. **(B,E)** Bast fibers control. **(C,F)** Bast fibers from the pulling side. **(D,G)** Bast fiber from the opposite side. The 817 cm^–1^ peak (positive contribution) was really ambiguous to assign to a particular polymer and was not considered as reliable.

In contrast the loading vector for PC2 (Figure 4A) showed both positive and negative contributions representing biological variability between the spectra of the three samples. This component explained 1.42% total variability. Major peaks making positive contributions in PC2 were assigned to lignin (red asterisk: 1,598 cm^–1^), cellulose/xyloglucan (black and green asterisks: 1,452 cm^–1^) based on the literature (Supplementary Table 1). Peaks making negative contributions were mainly assigned to cellulose (black asterisk: 379; 435; 457; 1,094; 1,118; 1,150; 1,376 cm^–1^), xylan/xyloglucan (green asterisk: 517; 1,094 cm^–1^). The corresponding PC2 score images (Figures 4B–G) indicate the relative spatial distribution of the positive and negative contributions. The majority of pixels making up the pulling side bast fiber wall image (Figure 4F) is colored in bright-dark red/black indicating that the corresponding cell walls are relatively enriched in lignin compared to either all of the control fiber cell wall layers (Figure 4E) and the S2/Gn secondary cell wall layers of opposite fibers (Figure 4G). Of particular interest is the presence of an intensely colored bright red band located at the junction between the two pulling side fiber cell walls indicating specific enrichment in this zone. In contrast, the majority of pixels making up all of the control fiber cell wall (Figure 4E) and the primary cell wall/S1 secondary cell wall of opposite fibers (Figure 4G) are green indicating that these cell wall layers are enriched in cellulose and potentially xyloglucan/xylan hemicelluloses compared to pulling side fibers. Taken together, the PC2 score images clearly indicate significant differences in the spatial distribution of cell wall polymers between these three fiber types.

**FIGURE 5:**
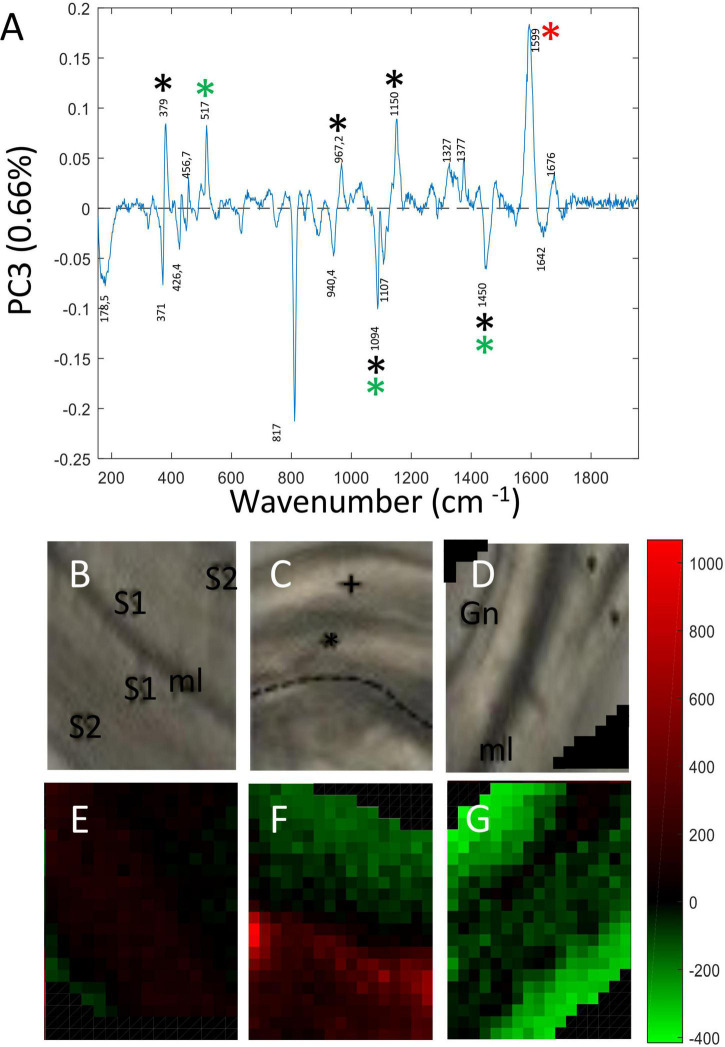
PC score imaging of PC3 (0.66%). **(A)** Loading vector of PC3. Peaks making positive contributions concerned chemical bonds related to cellulose (379; 1,150 cm^–1^), xyloglucan (517 cm^–1^), and lignin/aromatics (1,599 cm^–1^). Negative contribution concerned cellulose/xyloglucan peaks (1,094; 1,452 cm^–1^) Only major contributions (positive or negative) are considered. Black asterisk: cellulose, Red asterisk: lignin, Green asterisk: xylan and xyloglucan. Blue asterisk: pectin. **(B,E)** Bast fibers control. **(C,F)** Bast fibers from the pulling side. **(D,G)** Bast fiber from the opposite side. The 817 cm^–1^ peak (negative contribution) was really ambiguous to assign to a particular polymer and was not considered as reliable.

Principal Component 3 (PC3) accounted for 0.66% total variability. As for PC2, the corresponding loading vector (Figure 5A) contained both positive and negative contributions indicating that the variability observed at this level was biological in origin. Major peaks making positive contributions to PC3 were assigned to cellulose (379; 1,150; 457 cm^–1^), xyloglucan (517 cm^–1^), lignin (1,598 cm^–1^) and β glucan (969 cm^–1^). Peaks making negative contributions were assigned cellulose/hemicellulose (1,094 cm^–1^). Examination of PC3 score images (Figures 5B–G) revealed more subtle differences in the relative spatial distribution of cell wall polymers both between and within the three fiber samples. In the pulling side fiber sample (Figure 5F), clear differences in polymer distribution could be seen between the cell wall of the hypertrophied upper fiber distinguished by green pixels and the cell wall of the lower fiber that presented a size similar to those of control and opposite fibers and was characterized by red pixels. As indicated by pixel color, the hypertrophied pulling fiber cell wall most closely resembled that of the opposite fiber cell wall while that of the smaller pulling side fiber most closely resembled that of the control fiber.

Principal components 4, 5 and 6 made only extremely low contributions to the total variability (0.29, 0.2 and 0.14%, respectively). Interpretation of the corresponding PC score images was difficult due to the fact that different peaks assigned to the same polymers made both positive and negative contributions in the corresponding loading vector (Supplementary Figure 2).

### Raman chemical imaging reveals differences in the spatial distribution of cellulose and hemicellulose polymers

PC score imaging of flax fibers generates images where statistically significant differences in cell wall composition are represented by differences in pixel color (green through red). However, since these differences in pixel color are due to the combined effect of all polymers generating significant peaks in the corresponding loading vectors, it is not possible to obtain spatial information on individual polymers.

We therefore performed Raman chemical imaging based on peak wavenumbers identified in the different PC loading vectors (Figures 4, 5 and Supplementary Table 1) and assigned to the following polymers: (1) cellulose (435, 457, 890–920, 1,150, 1,376, and 1,439–1,491 cm^–1^); (2) hemicelluloses (517, 1,094, and 1,118 cm^–1^); and (3) lignin (1,270 and 1,539–1,720 cm^–1^). Although other peaks had been identified in the PC score image loading vectors, their intensities in the original Raman spectra were either too low and/or they could not be sufficiently discriminated from other peaks to allow accurate imaging.

**FIGURE 6:**
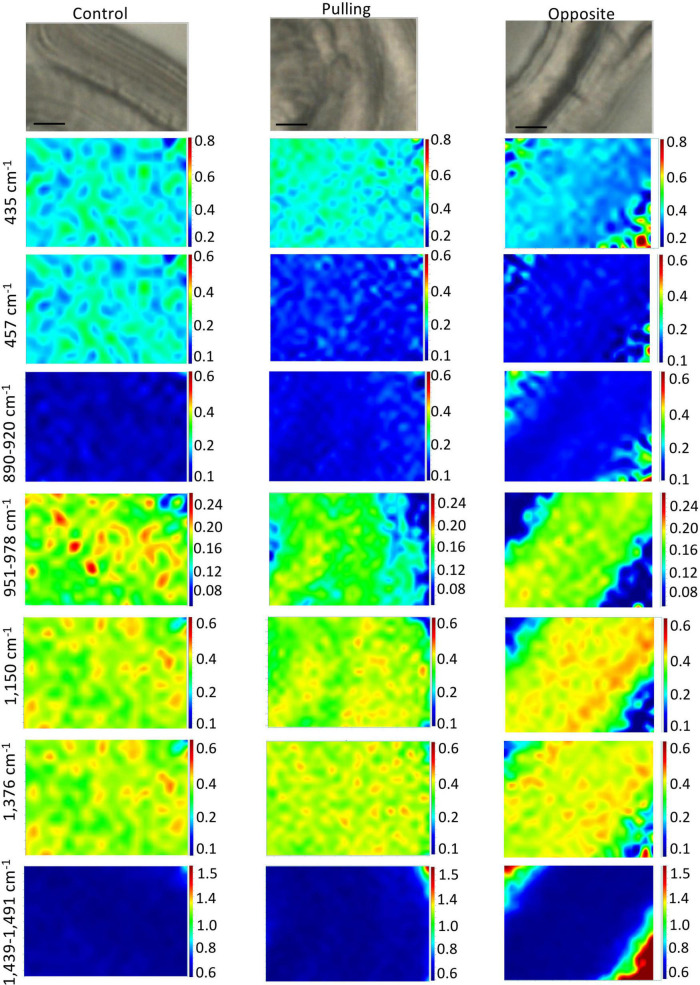
Raman chemical cartography of ratio (cellulose band/reference band 379 cm^–1^): major contributions of cellulose (435; 457; 890–920; 1,150; 1,376 cm^–1^) and cellulose/xyloglucan (1,439–1,491 cm^–1^). Color scale bar indicates pixel intensity for the imaged peak (red = high, blue = low). Within one peak, the color scale intensities were the same for each condition.

The chemical imaging for cellulose peak intensity ratios based on the 379 cm^–1^ reference peak are shown in Figure 6 (total peak intensities are given in Supplementary Figure 3). Overall, these results indicate that the gravitropic stress induces changes in cellulose distribution and/or relative intensity depending upon the band and fiber type (pulling, opposite) compared to control fibers. At 435 cm^–1^, a slight decrease of relative intensity is observed in the cell wall of opposite fibers, but not pulling-side fibers compared to the control. At 457 cm^–1^, a decrease is observed in the cell walls of both pulling- and opposite-side fibers compared to the control. In contrast the 890–920 cm^–1^ images (corresponding to the 900 cm^–1^ peak) are very similar for the three fiber types. For the 951–978 cm^–1^ band (corresponding to the 969 cm^–1^ peak), a reduction in pixel intensity is observed for both pulling- and opposite-side fibers. Images of the 1,150 and 1,376 cm^–1^ peaks show a very similar pattern with a reduction of pixel intensity in pulling side fibers compared to control fibers. In contrast, the cell walls of opposite fibers are characterized by relatively high intensity microdomains organized into parallel bands as opposed to the more disperse organization observed in both control and pulling fibers. The 1,439–1,491 cm^–1^ images (corresponding to the 1,452 cm^–1^ peak) are very similar for the three fiber types. The apparent increase/decrease of pixel ratio intensity observed in the top left-hand and bottom right-hand corners of opposite fiber images is most likely an artifact resulting from the fact that ratios are calculated on intensities acquired in the fiber lumen as indicated by low individual band intensities (Supplementary Figure 3).

**FIGURE 7:**
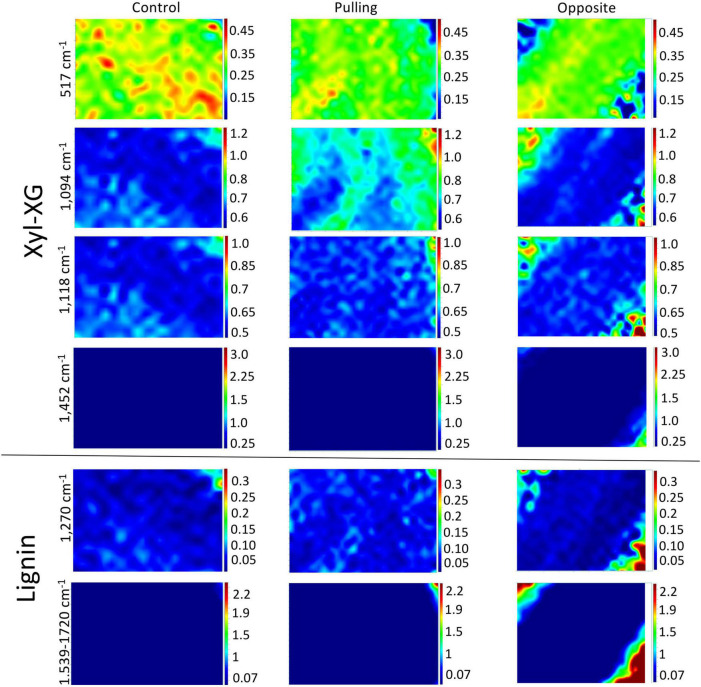
Raman chemical cartography of ratio (major band/reference band 379 cm^–1^): major contributions of xyloglucan-XG (517; 1,094; 1,452 cm^–1^), xylan-X (1,118 cm^–1^), lignin (1,270; 1,539–1,720 cm^–1^). Color scale bar indicates pixel intensity for the imaged peak (red = high, blue = low). Within one peak, the color scale intensities were the same for each condition.

The chemical imaging for hemicellulose and lignin peak intensity ratios based on the 379 cm^–1^ reference peak are shown in Figure 7 (total peak intensities are given in Supplementary Figure 4). At 517 cm^–1^, a decrease in pixel intensity can be observed in the walls of both pulling- and opposite-side fibers compared to control fibers. In addition, the relatively high intensity domains show a more linear organization in the walls of opposite fibers as compared to control fibers where the organization is more disperse. As was the case for the cellulose images, the top left-hand and bottom right-hand corners of opposite fiber images most likely represent artifacts. The 1,094 cm^–1^ images for opposite fibers are very similar to those for the control fibers. In contrast, pulling-side fibers are characterized by alternating linear bands of high and low intensity pixels as compared to the regular (low intensity) organization of control and opposite fibers.

Lignin chemical images (1,270 and 1,539–1,720 cm^–1^) are globally very similar for all three fiber types. Nevertheless, relatively higher intensity pixels appear to be organized into discrete linear bands in the cell walls of pulling-side fibers compared to both control and opposite side fibers.

## Discussion

Flax is an economically important dual-purpose species cultivated for both its cellulose-rich bast fibers and for its seeds rich in unsaturated fatty acids. Like a number of other annual crops this plant is subjected to lodging in extreme weather conditions complicating harvest and leading to reduced fiber yields (Goudenhooft et al., 2019). Provided that adverse climatic conditions do not persist, lodged flax stems are able to recover to a vertical position and continue normal growth.

The ability of plants in general to return to the vertical position is believed to involve two tropisms: (i) gravitropism, the response to inclination and (ii) autotropism, the response to stem curvature (Moulia et al., 2021). In both cases, the plant’s response involves “sensing mechanisms” that detect the deviation from the correct position, and “motor processes” responsible for the recovery of the stem to the vertical position. Different motor mechanisms are used depending upon the nature of the plant: in annual non-woody plants such as *Arabidopsis*, the return to the correct position is achieved by differential growth of the floral stem (Bastien et al., 2015). In this case, auxin promotes elongation growth in the lower side of the stem leading to upwards curvature. In woody plants such as poplar, stem recovery is mediated via the production of specialized reaction wood characterized by the presence of cellulose-rich G-(gelatinous) layer that generate tension forces (Mellerowicz and Gorshkova, 2012; Chang et al., 2014).

Given the structural similarity of flax bast fibers and wood G-layer, it also seems likely that the former may play a role in redressing inclined flax stems. A recent study (Ibragimova et al., 2017) showed that the stems of flax plants were able to recover to the vertical position after 4 days when pots were tilted through 90°. Analyses showed that the recovery was associated with the formation of G-layer in flax xylem tissue and important modifications in the morphology of bast fibers on the pulling (upper) side of stems. Our measurements of several different morphometric parameters of fibers from control plants and tilted plants revealed statistically significant differences between pulling side fibers and both control fibers and opposite side fibers. As previously observed (Ibragimova et al., 2017) pulling side fibers were characterized by a larger surface area and thinner cell wall reflecting profound changes in the cell wall developmental program. While opposite side fibers were morphologically more similar to normal fibers for all parameters evaluated, analyses revealed that the differences observed were also statistically significant. These results are in agreement with many comparative analyses of tension wood, opposite wood and normal wood that have shown that leaning not only results in modifications to the pulling side of the inclined stem compared to normal wood, but also to the opposite side (Pilate et al., 2004; Gorshkova et al., 2010; Schreiber et al., 2010; Mellerowicz and Gorshkova, 2012; Felhofer et al., 2020).

Observed differences in the TBO coloration of fiber cell walls suggested that the imposed stress not only affected fiber morphology, but also cell wall composition (O’Brien et al., 1964) in agreement with the observations of Beaugrand et al. (2022) who compared bast fiber FT-IR spectra from control and tilted flax. In order to characterize further such modifications, we used confocal Raman spectroscopy. This type of vibrational spectroscopy has been long established as a powerful technique for identifying plant cell wall spectratypes and has been successfully used to characterize the cell wall polymers in woody plants such as spruce, beech and poplar as well as in herbaceous plants such as *Trifolium* (Gierlinger et al., 2008, 2012; Schreiber et al., 2010; Gorzsás et al., 2011; Prats Mateu et al., 2016; Gorzsás, 2017; Felhofer et al., 2020). While both Raman and Fourier Transform Infra-Red (FT-IR) spectroscopy techniques can be used to study plant cell walls, the higher spatial resolution of Raman spectroscopy (0.3–1 μm) compared to FT-IR 2–20 μm) enables analysis at the level of different cell wall layers to be undertaken (Lasch and Naumann, 2006; Simon et al., 2018).

A comparison of fibers from tilted plants (pulling-/opposite-side) and control plants using PC score imaging provided detailed information about modifications in cell wall polymer composition induced by gravitational stress. While the first PC explained 92,31% total variance, the corresponding loading vector was made up of (a) peaks that only made positive contributions and (b) were very similar to those present in the fiber average spectra. These observations would suggest that PC1 most likely corresponds to an overall variation in the spectrum resulting from physical variations such as laser intensity or small differences in the surface flatness of the sections.

This result would suggest that the observed differences in fiber morphology result mainly from changes to the spatial distribution of and/or interaction between polymers, rather than any major changes in biosynthesis. Certainly, such a hypothesis is in agreement with the average spectra of the three fiber samples that, while showing clear differences in the height of some peaks, do not present the appearance/disappearance of peaks. Proteomics of the fiber-bearing outer tissues of flax stems identified a large number of cell-wall Glycosyl Hydrolase (GH) enzymes that would be capable of such modifications (Chabi et al., 2017). Nevertheless, data obtained from PC2 – PC6 loading vectors and images highlighted small, but significant differences in the relative enrichment/depletion, as well as in the spatial distribution of a number of different polymers within fiber cell wall layers. Overall, pulling side fibers appeared to be relatively enriched in lignin but were depleted in cellulose and hemicellulose compared to fibers from control plants. While opposite fibers showed enrichment/depletion of the same polymers observed in pulling side fibers, these same polymers were selectively enriched/depleted in different cell wall layers.

However, PC score images reveal statistically significant differences based on the constitutive peaks making positive/negative contributions to the loading vector. Since, several peaks, often indicative of different polymers, usually contribute to the loading vectors, such images do not provide information about the spatial distribution of each polymer. In order to obtain such information, chemical images were generated based upon the different significant peaks identified in the vector loadings.

Overall, the obtained images show that discrete changes occur not only in the relative intensity of certain bands assigned to cellulose, hemicelluloses and lignin, but also in their organization within the cell wall. Depending upon the band and fiber type considered, the plant’s response to the gravitropic stress appears to involve the transition from a disperse microdomain organization toward an organization in linear bands. It can be hypothesized that these localized changes in the composition and distribution of polymers within the different cell wall layers contribute to the observed changes in stressed fiber morphology and the associated modifications in mechanical properties necessary to return the stem to a vertical position (Clair et al., 2018; Gorshkova et al., 2018).

## Conclusion

Our results show that gravitropic stress in flax induces discrete but significant changes in the composition and spatial organization of cell wall polymers in both pulling side and opposite side fibers compared to control fibers. These observations underline the importance of using *in situ* imaging techniques for a more thorough understanding of cell wall biology. In this context, Raman vibrational microscopy coupled with multivariate PC score chemical imaging represents a powerful tool for investigating cell wall dynamics. The use of alternative multivariate analytical approaches based on cluster analysis, vertex component analysis or non-negative matrix factorization (Gierlinger, 2018) could also help to decipher the complex modifications that occur in plant cell walls in response to gravitropic stress.

## Data availability statement

The original contributions presented in this study are included in the article/Supplementary material, further inquiries can be directed to the corresponding author.

## Author contributions

A-SB, MM, and SH conceptualized the manuscript and wrote the manuscript. LD analyzed the data through MATLAB software, performed PCA analysis, and PC loadings (chemometric statistics). A-SB and SH interpreted the results and wrote the revised version of the manuscript. A-SB illustrated the figures. AD programmed the R-interface and undertook the morphometric data analysis (morphometric statistics). All authors discussed the results, read, and approved the manuscript.

## Footnotes

1
https://imagej.net/software/fiji/

## Supplementary material

Supplementary Figure 1 Bast fiber phenotypes observed by light microscopy. **(A)** Control BF exhibits layered cell walls and thin gap could be observed (black arrow) in the middle lamella location or in a three-way junction. **(B)** Opposite side BF: larger gaps were observed but global normal phenotype is still present. **(C,D)** Altered pulling BF: folding of the thin cell wall could be observed in restricted area of the cell wall (black arrow, **C**), but in some BF, all cell wall seems detached from the neighbored BF causing a real isolation **(D)**. Bar = 10 μm.

Supplementary Figure 2 PC Score imaging according to PC4 (0.29%), PC5 (0.2%) and PC6 (0.13%). **(A–C)**: Video captures. **(B)**: Black dotted line corresponds to the middle lamella. *, + symbolize the two parts of S2-S2/Gn cell walls that are separating due to the treatment. PC loadings: black asterisk: cellulose-, green asterisk: xylan/xyloglucan-, red asterisk: lignin/aromatics- related peaks. **(D–F)**, PC4: Control bast fibers showed black pixels. In pulling side fibers, an enrichment was observed in cellulose (379; 1,150 cm^–1^), xyloglucan/xylan (517 cm^–1^). In opposite fibers, walls are enriched in cellulose (435; 1,376 cm^–1^), xyloglucan/xylan (1,094; 1,126 cm^–1^), and lignin/aromatics (1,599 cm^–1^). **(G–I)**, PC5: Red pixels indicate that the cell wall is rich in cellulose (435 cm^–1^), xyloglucan/xylan (1,094 cm^–1^), and lignin/aromatics (1,270 cm^–1^). For green pixels, walls are enriched in cellulose (379; 398; 900; 1,150 cm^–1^), xyloglucan/xylan (1,452 cm^–1^). **(J–L)**, PC6: Red pixels indicate that the cell wall is rich in cellulose (405; 1,150 cm^–1^), xyloglucan/xylan (517 cm^–1^). The 817 cm^–1^ peak (positive or negative contribution) was ambiguous to assign and it was not considered as reliable.

Supplementary Figure 3 Raman chemical imaging of major cellulose-related peaks, in intensities. Major cellulose positive or negative contributions in PC loadings were only chosen (379; 435; 457; 1,094; 1,150; 1,376; 1,439–1,491 cm^–1^). Compared to the control, spatial reorganization is observed in both stressed fibers. In pulling side, gaps between layers are seen as layers bent. Close to this curvature a decrease in cellulose is observed. Some peaks are confined into middle micro-domains (pockets) as pearl necklace. For opposite side, symmetric homogenous and regular layers are observed on each side from the middle lamella. Such micro-domains, or continuous layers, concentrated 379; 435; 1,150; and 1,376 cm^–1^ chemical bonds.

Supplementary Figure 4 Raman chemical imaging of major xylan/xyloglucan- and lignin-related peaks, in intensities. Major positive or negative contributions in PC loadings were only chosen: xylan/xyloglucan and cellulose (517; 1,118; 1,452 cm^–1^), and, aromatics/lignin (1,270; 1,539–1,720 cm^–1^). Within one peak, the color scale intensities were the same for each condition. Control fibers exhibits symmetric deposition from each side of the middle lamella with a progressive increase (yellow then orange) with enriched scattered zones (dark orange to red pixels). In pulling fibers, spatial deposition is similar to a pearl necklace, enriched in 517, 1,094, and 1,118 cm^–1^ chemical bonds. As bast fibers are considered as hypolignified (control), stress ones exhibited a clear increase of chemical bonds related to aromatics (polymerized lignin was not established with phloroglucinol-HCl staining). Spatial distribution shows layers.

Supplementary Table 1 Assignment of Raman bands in the range of 300:1,800 cm^–1^ to functional groups of bast fibers polymers. Average spectra peaks and PC loadings ones (only for major contributions) were used to establish this table.
