# Supplementary figures and images for: Raman spectroscopy mapping of changes in the organization and relative quantities of cell wall polymers in bast fiber cell walls of flax plants exposed to gravitropic stress
Source: Front Plant Sci. 2022 Aug 22;13:976351. doi: 10.3389/fpls.2022.976351 (PMC9442035; doi:10.3389/fpls.2022.976351)

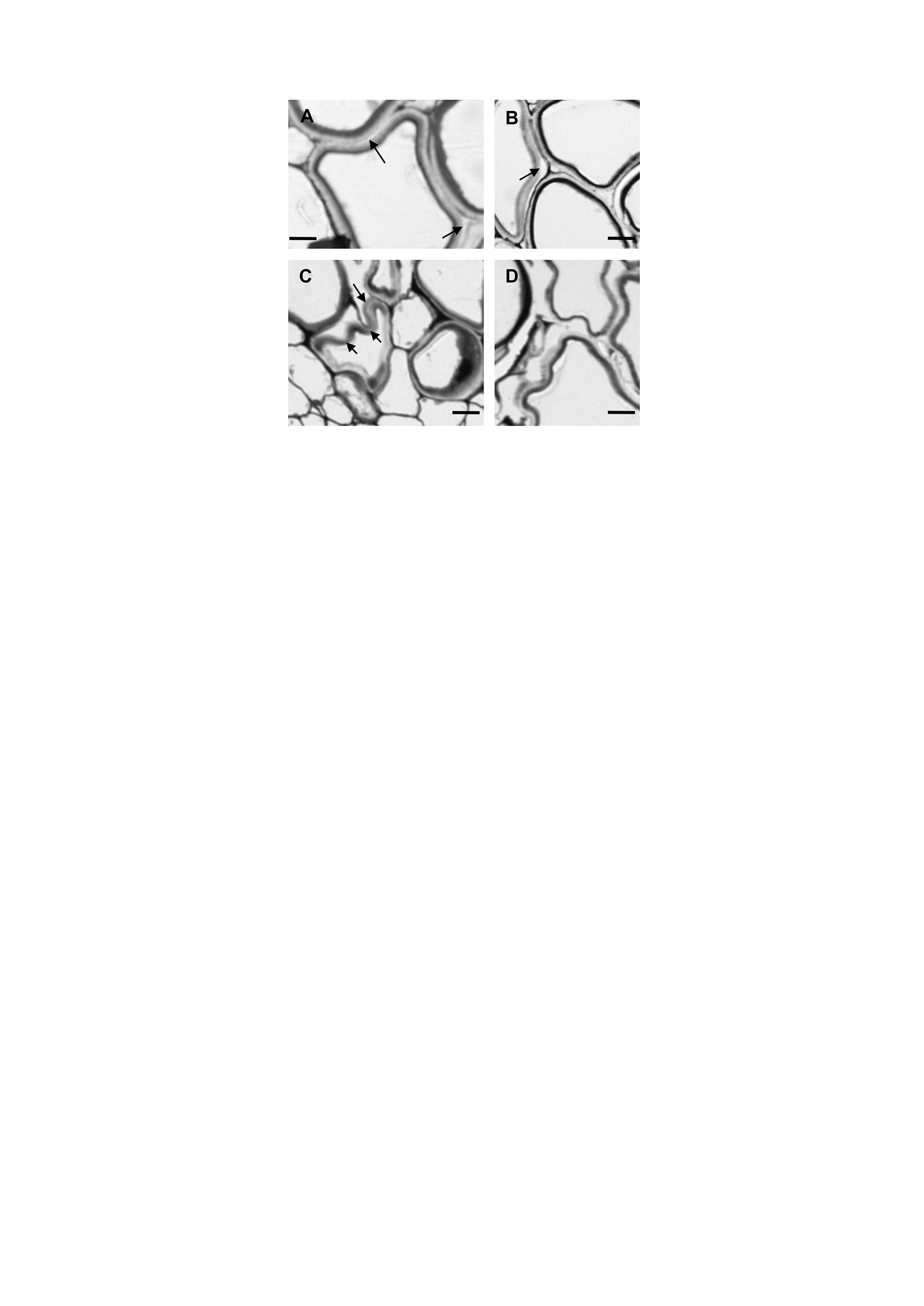

Supplement: Supplementary Figure 1 — Bast fiber phenotypes observed by light microscopy. (A) Control BF exhibits layered cell walls and thin gap could be observed (black arrow) in the middle lamella location or in a three-way junction. (B) Opposite side BF: larger gaps were observed but global normal phenotype is still present. (C,D) Altered pulling BF: folding of the thin cell wall could be observed in restricted area of the cell wall (black arrow, C), but in some BF, all cell wall seems detached from the neighbored BF causing a real isolation (D). Bar = 10 μm. [file Image_1.TIF]

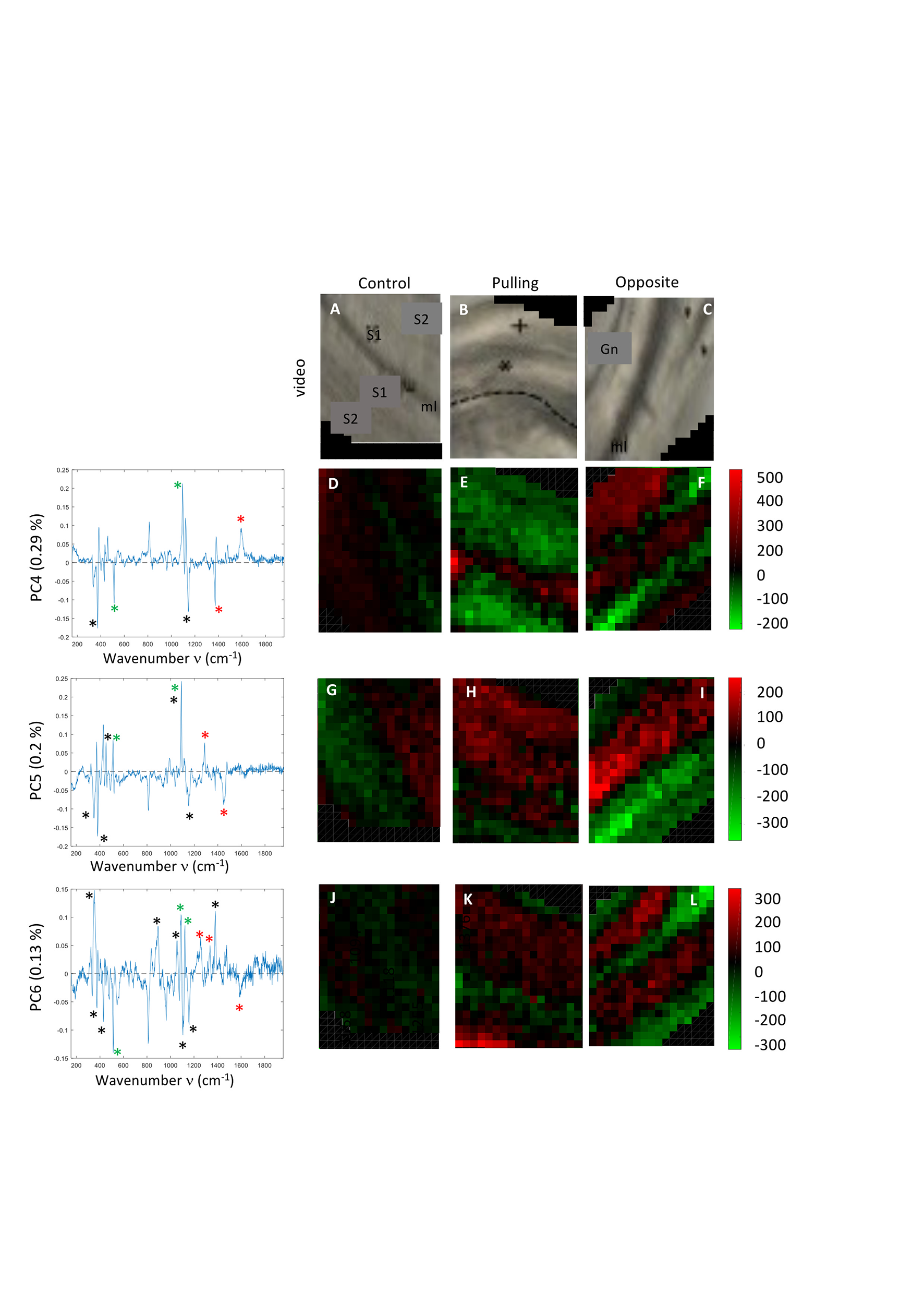

Supplement: Supplementary Figure 2 — PC Score imaging according to PC4 (0.29%), PC5 (0.2%) and PC6 (0.13%). (A–C): Video captures. (B): Black dotted line corresponds to the middle lamella. *, + symbolize the two parts of S2-S2/Gn cell walls that are separating due to the treatment. PC loadings: black asterisk: cellulose-, green asterisk: xylan/xyloglucan-, red asterisk: lignin/aromatics- related peaks. (D–F), PC4: Control bast fibers showed black pixels. In pulling side fibers, an enrichment was observed in cellulose (379; 1,150 cm–1), xyloglucan/xylan (517 cm–1). In opposite fibers, walls are enriched in cellulose (435; 1,376 cm–1), xyloglucan/xylan (1,094; 1,126 cm–1), and lignin/aromatics (1,599 cm–1). (G–I), PC5: Red pixels indicate that the cell wall is rich in cellulose (435 cm–1), xyloglucan/xylan (1,094 cm–1), and lignin/aromatics (1,270 cm–1). For green pixels, walls are enriched in cellulose (379; 398; 900; 1,150 cm–1), xyloglucan/xylan (1,452 cm–1). (J–L), PC6: Red pixels indicate that the cell wall is rich in cellulose (405; 1,150 cm–1), xyloglucan/xylan (517 cm–1). The 817 cm–1 peak (positive or negative contribution) was ambiguous to assign and it was not considered as reliable. [file Image_2.TIF]

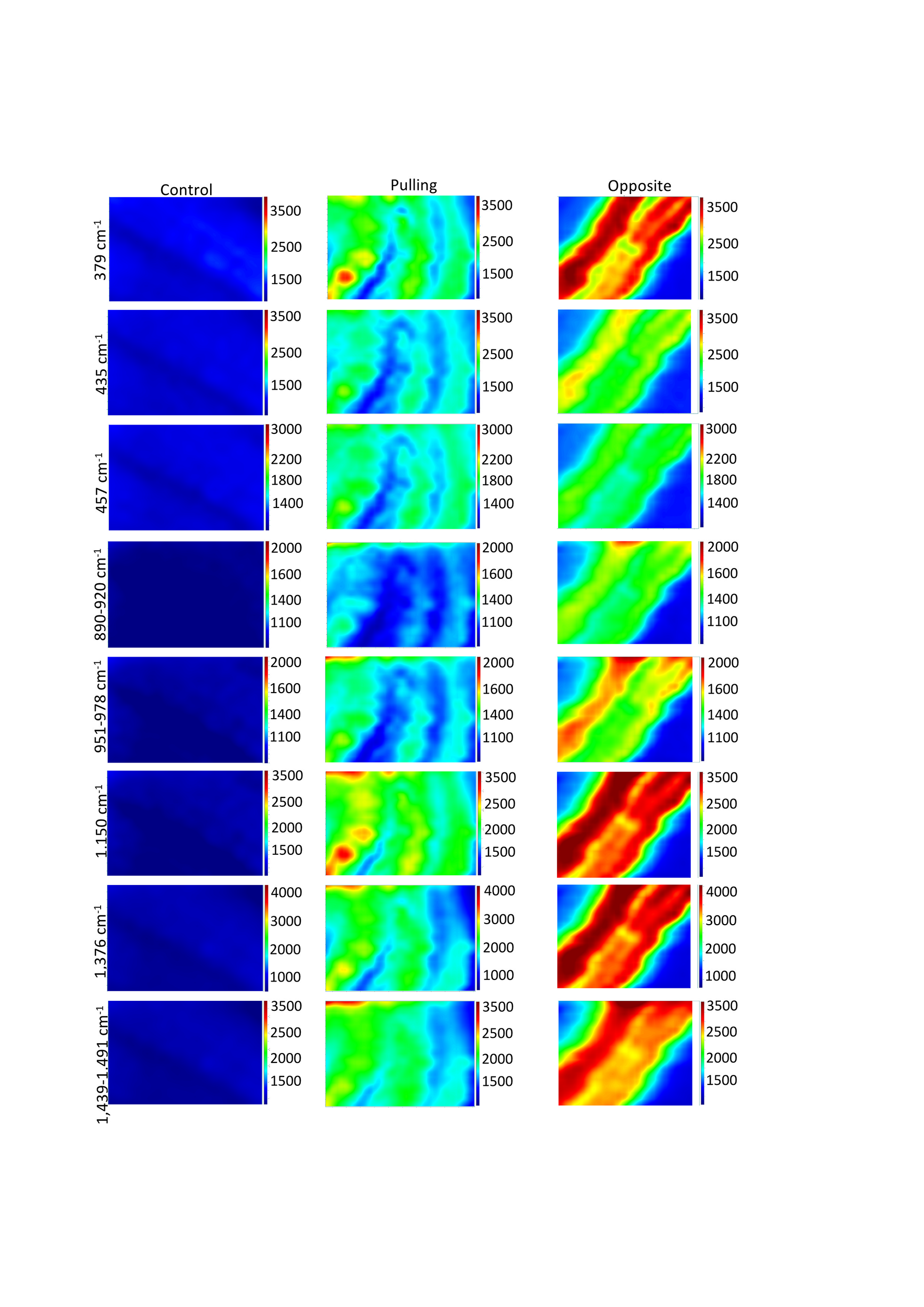

Supplement: Supplementary Figure 3 — Raman chemical imaging of major cellulose-related peaks, in intensities. Major cellulose positive or negative contributions in PC loadings were only chosen (379; 435; 457; 1,094; 1,150; 1,376; 1,439–1,491 cm–1). Compared to the control, spatial reorganization is observed in both stressed fibers. In pulling side, gaps between layers are seen as layers bent. Close to this curvature a decrease in cellulose is observed. Some peaks are confined into middle micro-domains (pockets) as pearl necklace. For opposite side, symmetric homogenous and regular layers are observed on each side from the middle lamella. Such micro-domains, or continuous layers, concentrated 379; 435; 1,150; and 1,376 cm–1 chemical bonds. [file Image_3.TIF]

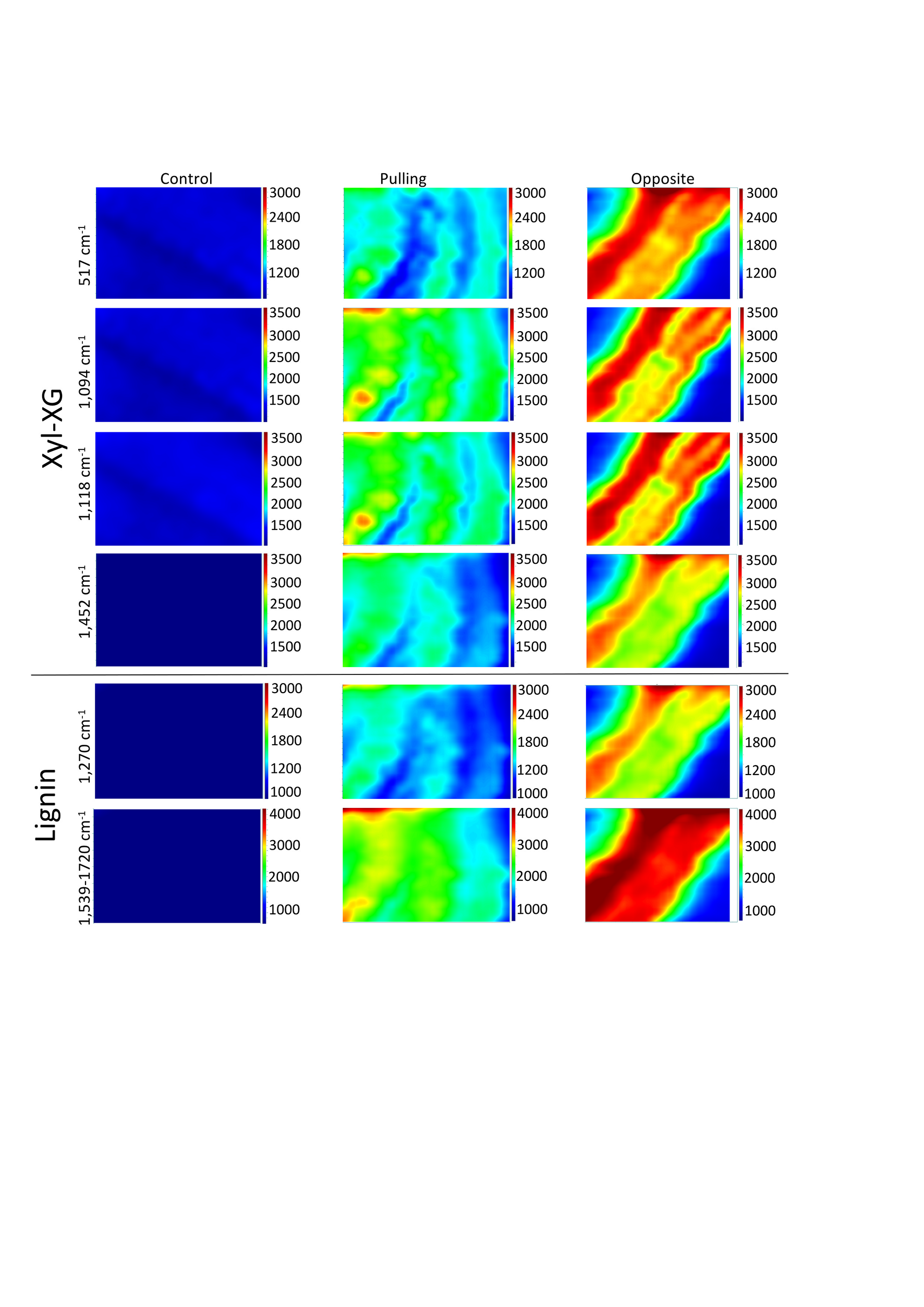

Supplement: Supplementary Figure 4 — Raman chemical imaging of major xylan/xyloglucan- and lignin-related peaks, in intensities. Major positive or negative contributions in PC loadings were only chosen: xylan/xyloglucan and cellulose (517; 1,118; 1,452 cm–1), and, aromatics/lignin (1,270; 1,539–1,720 cm–1). Within one peak, the color scale intensities were the same for each condition. Control fibers exhibits symmetric deposition from each side of the middle lamella with a progressive increase (yellow then orange) with enriched scattered zones (dark orange to red pixels). In pulling fibers, spatial deposition is similar to a pearl necklace, enriched in 517, 1,094, and 1,118 cm–1 chemical bonds. As bast fibers are considered as hypolignified (control), stress ones exhibited a clear increase of chemical bonds related to aromatics (polymerized lignin was not established with phloroglucinol-HCl staining). Spatial distribution shows layers. [file Image_4.TIF]
