# Supplementary material for: Raman spectroscopy mapping of changes in the organization and relative quantities of cell wall polymers in bast fiber cell walls of flax plants exposed to gravitropic stress
Source: Front Plant Sci. 2022 Aug 22;13:976351. doi: 10.3389/fpls.2022.976351 (PMC9442035; doi:10.3389/fpls.2022.976351)
Supplement: Supplementary Table 1 — Assignment of Raman bands in the range of 300:1,800 cm–1 to functional groups of bast fibers polymers. Average spectra peaks and PC loadings ones (only for major contributions) were used to establish this table. [file Table_1.pdf]

Polymers	Expermimental measures cm^{-1}	Literature cm^{-1}	Assignment	References
Cellulose	1478 w	1478 m	δ(CH2) scissors	(Agarwal and Ralph 1997; Kavkler and Demšar 2011)
	1459 w	1455	δ(CH2)	(Agarwal and Ralph 1997; Gierlinger 2014)
	1407 mw	1407-1408 w	δ(CH2)	(Agarwal and Ralph 1997; Edwards, Farwell, and Webster 1997; Kavkler and Demšar 2011)
	1376 m	1378-1379 s-1380	δ(CH2)	(Agarwal and Ralph 1997; Bonizzoni et al. 2016; Kavkler and Demšar 2011; Agarwal et al. 2019)
	1339 w	1336- 1337-1338-1339 -1340 m	OH in plane bending	
	1233 w		OH in plane bending	(Gierlinger et al. 2008; Zhang et al. 2017)
	1210 w			
	1192			
	1150 s	1147-1150-1152-1154 m	ν(CC), ν(CO) asymmetric, ring breathing (glucopyranose)	(Bonizzoni et al. 2016; Gierlinger 2014; Kavkler and Demšar 2011; Agarwal et al. 2019; Szymańska-Chargot et al. 2016)
	1094 s	1092-1095-1096 s	ν(COC) asymmetric, glycosidic ring breathing, skeletal	
	1014 w			(Kavkler and Demšar 2011; Agarwal and Ralph 1997; Bonizzoni et al. 2016)
	990 w	993-995-997-999 w	$\rho(CH_2)$	
	969 m	966-968-971-974-980 w	$\rho(CH_2)$ skeletal β glucan	
	912 m		δ(COC), symmetric	(Edwards, Farwell, and Webster 1997)
	900 m	893-900-914 mw	δ(HCC), δ(HCO) cluster of peaks-methine bending	(Agarwal and Ralph 1997; Kavkler and Demšar 2011)
	607	602-607-609-611 w	δ(CCH)	
	599 w			(Zhang, Chen, and Xu 2017)
	562 w	565-567-575 w	δ(COC), ring	(Agarwal and Ralph 1997; Agarwal et al. 2019; Kavkler and Demšar 2011; Bonizzoni et al. 2016)
	490 m	489-490-492-496 w	δ(COC), glycosidic linkage, xylan	
	435 m	434-435-437 m	δ(COC), δ(CCC), ring deformation	
	405 w			
	379 s	377-379-380-382 ms	δ(CCC), symmetric ring deformation	
	347 w	345-347-350-351 mw	δ(CCC), ring deformation	

	331 sh	329-330-331-333 mw	δ(CCC), ring deformation, skeletal bending	
Xylan	1729	1725-1745	C=O of acetyl or caroxylic acid group	(Gierlinger et al. 2008; Gierlinger 2014)
Xyloglucan	1459 w	1460	δ(CH2) symmetric bending on pyranose ring	
Xylan Arabinoxylan	1118 s	1121	ν(COC) symmetric, glycosidic ring breathing	(Gierlinger et al. 2008; Gierlinger 2014; Bonizzoni et al. 2016; Piot, Autran, and Manfail 2001)
Xyloglucan Arabinoxylan	1094 s	1091-1094	xyloglucan β (1-4) linked glucose	(Agarwal and Ralph 1997; Szymańska-Chargot et al. 2016; Piot, Autran, and Manfail 2001)
Xyloglucan	517 m	517-518-519-521 m	δ(COC), glycosidic linkage/CCC ring deformation	(Agarwal and Ralph 1997; Agarwal et al. 2019; Kavkler and Demšar 2011; Bonizzoni et al. 2016)
Xylan	490 m	489-490-492-494-496 w	δ(COC), glycosidic linkage, xylan	
Xylan	457 m	457-458-460	δ(COC), δ(CCC), ring deformation, xylan	
Lignin	1658 mw	1657-1660	δ(CC), C=O coniferyl aldehyde, C=C coniferyl alcohol	(Gierlinger 2014)
	1620 s	1621	δ(CC)	
	1599 w	1593-1601 m	ν(C=C), aromatics	(Gierlinger et al. 2008; Gierlinger 2014)
	1506 w		Aryl ring stretch	(Agarwal and Ralph 1997; Prats Mateu et al. 2016)
	1452 w	1453	CH_3 scissoring	
	1421 w		Lignin methoxy deformation	
	1363 sh		Ring deformation in plane COH bending	
	1332 m	1331-134	Syringil, Aliphatic –OH stretch	(Gierlinger 2014)
	1294 w	1292-1293-1294 mw	δ(CH2) twisting, long chain hydrocarbon	(Agarwal and Ralph 1997; Prats Mateu et al. 2016; Kavkler and Demšar 2011)
	1270 m		Aryl-O stretching of aryl-OH and aryl-O-CH_3 (G unit)	
	1136 s		Coniferyl/sinalpyladehyde	(Gierlinger 2014)
	1058 w	1057-1058-1065w	ν(CO) , 1°alcohol group	(Agarwal and Ralph 1997; Prats Mateu et al. 2016; Kavkler and Demšar 2011)
	1031 w	1034-1035-1037 w	ν(CC), ν(CO) 2° alcohol group	
	925 w			(Agarwal and Ralph 1997)
	371 sh, vw	370	Syringil unit	(Agarwal et al. 2019)

Pectin	[840-860]	850-854	Include esterified and acetylated pectin	(Szymańska-Chargot et al. 2016; Synytsya et al. 2003; Pan, Pu, and Sun 2017)

Anotations:
s : strong, *ms* : medium to strong, *m* : medium, *mw* : medium weak, *sh* : shoulder, *w* : weak, *vw*: very weak

ν stretching, δ bending, ρ rotating

References cited in this Supplementary Table 1:

Agarwal, U. P., and Ralph, S. A. (1997). FT-Raman spectroscopy of wood: Identifying contributions of lignin and carbohydrate polymers in the spectrum of black spruce (Picea mariana). *Appl. Spectrosc.* 51, 1648–1655. doi:10.1366/0003702971939316

Agarwal, U.P., Ralph, S.A., Padmakshan, D.L., and Foster, C.E. (2019). Estimation of Syringyl Units in Wood Lignins by FT-Raman Spectroscopy. *J. Agric. Food Chem.* 67(15): 4367–74. doi:10.1021/acs.jafc.8b06707.

Ageeva, M. V., Petrovská, B., Kieft, H., Sal'nikov, V.V., Snegireva, A.V., Van Dam, J.E.G., Van Veenendaal, W.L.H., Emons, A.M.C., Gorshkova, T.A., and Van Lammeren, A.A.M. (2005). Intrusive Growth of Flax Phloem Fibers Is of Intercalary Type. *Planta* 222 (4): 565–74. https://doi.org/10.1007/s00425-005-1536-2

Bonizzoni, L., Bruni, S., Fanti, G., Tiberio, P., and Zaffino, C. (2016). Ageing of flax textiles: Fingerprints in micro-Raman spectra of single fibres. *Microchem. J.* 125, 69–74. doi:10.1016/j.microc.2015.11.011

Edwards, H.G., Farwell, D.W., and Webster, D. (1997). FT Raman Microscopy of Untreated Natural Plant Fibres. *Spectrochim. Acta A. Mol. Biomol. Spectrosc.,* 53A, 2383–92. doi.org/10.1016/S1386-1425(97)00178-9.

Gierlinger, N. (2014). Revealing changes in molecular composition of plant cell walls on the micron-level by Raman mapping and vertex component analysis (VCA). *Front. Plant Sci.* 5, 1–10. doi:10.3389/fpls.2014.00306

Gierlinger, N., Goswami, L., Schmidt, M., Burgert, I., Coutand, C., Rogge, T., et al. (2008). In situ FT-IR microscopic study on enzymatic treatment of poplar wood cross-sections. *Biomacromolecules* 9, 2194–2201. doi:10.1021/bm800300b

Kavkler, K., and Demšar, A. (2011). Examination of Cellulose Textile Fibres in Historical Objects by Micro-Raman Spectroscopy. *Spectrochim. Acta. A. Mol. Biomol. Spectrosc.,* 78, 740–46. doi.org/10.1016/j.saa.2010.12.006.

Pan, T.T., Pu, H. and Sun, D.W. (2017). Insights into the Changes in Chemical Compositions of the Cell Wall of Pear Fruit Infected by Alternaria Alternata with Confocal Raman Microspectroscopy. *Postharvest Biol. Technol.* 132(June): 119–29. http://dx.doi.org/10.1016/j.postharvbio.2017.05.012.

Piot, O., Autran, J.C. and Manfail, M. (2001). Investigation by Confocal Raman Microspectroscopy of the Molecular Factors Responsible for Grain Cohesion in the Triticum Aestivum Bread Wheat. Role of the Cell Walls in the Starchy Endosperm. *J. Cereal Sci.* 34(2): 191–205. Doi: 10.1006/jcrs.2001.0391

Prats Mateu, B., Hauser, M.T., Heredia, A., and Gierlinger, N. (2016). Waterproofing in Arabidopsis: Following Phenolics and Lipids In Situ by Confocal Raman Microscopy. *Front. Chem.,* 4, 10. doi.org/10.3389/fchem.2016.00010.

Synytsya, A., Čopíková, J., Matějka, P., and Machovič, V. (2003). Fourier transform Raman and infrared spectroscopy of pectins. *Carbohydr. Polym.* 54, 97-106. doi.org+110/1016/S0144-8617(03)00158-9

Szymańska-Chargot, M., Chylińska, M., Pieczywek, P. M., Rösch, P., Schmitt, M., Popp, J., and Zdunek, A. (2016). Raman imaging of changes in the polysaccharides distribution in the cell wall during apple

fruit development and senescence. *Planta* 243, 935–945. doi:10.1007/s00425-015-2456-4

Zhang, X., Chen, S., Ramaswamy, S., Kim, Y. S., and Xu, F. (2017). Obtaining pure spectra of hemicellulose and cellulose from poplar cell wall Raman imaging data. *Cellulose* 24, 4671-4682. doi.org/10.1007/sf0570-017-1486-4.
